# Supplementary material for: The Systems Biology Research Tool: evolvable open-source software
Source: BMC Syst Biol. 2008 Jun 29;2:55. doi: 10.1186/1752-0509-2-55 (PMC2446383; doi:10.1186/1752-0509-2-55)
Supplement: Additional file 1 — SBRT Archive. An archive of the current version of the Systems Biology Research Tool. [file 1752-0509-2-55-S1.zip › sbrt-1.4.0/doc/developers_guide/api/sbrt/kernel/fba/comp/FluxCvComputer.html]

FluxCvComputer


|  |  |  |
| --- | --- | --- |
| **Overview** \| **Package** \| **Class** \| **Use** \| **Tree** \| **Deprecated** \| **Index** \| **Help** | |  |
| **PREV CLASS**   **NEXT CLASS** | **FRAMES**    **NO FRAMES**     **All Classes** |  |
| SUMMARY: NESTED \| FIELD \| CONSTR \| METHOD | DETAIL: FIELD \| CONSTR \| METHOD |  |


---


## sbrt.kernel.fba.comp Interface FluxCvComputer

**All Known Subinterfaces:**: FluxIntervalComputer

**All Known Implementing Classes:**: AbstractCvComputer, AbstractFluxIntervalComputer, CdHarFluxVectorComputer, FastMsFluxRangeComputer, FbaOptComputer, InitialPointComputer, MsFluxIntervalComputer, ReverseRxnFluxIntervalComputer, RxnDeletionComputer, SimpleFluxIntervalComputer

---

``` public interface FluxCvComputer ```

This interface is used to represent a flux constraint variation computer.
A constraint-variation computer is constructed relative to a particular
fluxome. It allows one to compute the effect of varying the constraints
within that fluxome.

**Author:**
:   This interface was written and documented by Jeremiah Wright
    while in the Wagner lab.

---

| **Method Summary** | |
| --- | --- |
| `Interval` | `getFluxConstraint(java.lang.String rxnName)`             Returns the current flux constraint on the reaction with the provided name. |
| `java.util.Map<java.lang.String,Interval>` | `getFluxConstraints()`             Returns all current flux constraints. |
| `Fluxome` | `getFluxome()`             Returns the fluxome used to construct this flux constraint-variation computer. |
| `Interval` | `getRxnExprConstraint(MathExpr<java.lang.String> rxnNameExp)`             Returns the current constraint on the provided expression. |
| `java.util.Map<? extends MathExpr<java.lang.String>,Interval>` | `getRxnExprConstraints()`             Returns all current flux expression constraints. |
| `void` | `restoreFluxConstraint(java.lang.String reactionName)`             Restores the flux constraint for the reaction with the provided name to the value contained in the fluxome. |
| `void` | `restoreFluxConstraints()`             Restores the flux constraints for all reactions in the fluxome to those values contained in the fluxome. |
| `void` | `restoreFluxConstraints(java.util.Collection<java.lang.String> reactionNames)`             Restores the flux constraints for the provided reaction names to the values contained in the fluxome. |
| `void` | `restoreRxnExprConstraint(MathExpr<java.lang.String> rxnNameExp)`             Restores the constraint on the provided flux expression to the value contained in the fluxome. |
| `void` | `restoreRxnExprConstraints()`             Restores the constraints on all flux expressions to the values contained in the fluxome. |
| `void` | `restoreRxnExprConstraints(java.util.Collection<? extends MathExpr<java.lang.String>> rxnNameExps)`             Restores the constraints on the provided expressions to the values contained in the fluxome. |
| `void` | `setFluxConstraint(java.lang.String reactionName, Interval c)`             Changes the flux constraint of the reaction with the provided name. |
| `void` | `setFluxConstraints(java.util.Map<java.lang.String,Interval> fluxConstraints)`             Changes the flux constraints on more than one reaction. |
| `void` | `setRxnExprConstraint(MathExpr<java.lang.String> e, Interval c)`             Changes the constraint on the provided mathematical expression of fluxes. |
| `void` | `setRxnExprConstraints(java.util.Map<? extends MathExpr<java.lang.String>,Interval> constraints)`             Changes the constraints on more than one mathematical expression of fluxes. |

**Method Detail**

### getFluxome

```
Fluxome getFluxome()
```

:   Returns the fluxome used to construct this flux constraint-variation
    computer.

    :   **Returns:**: the fluxome used to construct this flux constraint-variation computer.

---


### getRxnExprConstraints

```
java.util.Map<? extends MathExpr<java.lang.String>,Interval> getRxnExprConstraints()
```

:   Returns all current flux expression constraints.

    :   **Returns:**: mathematical expressions of reaction names mapped to their respective constraint.

---


### getFluxConstraints

```
java.util.Map<java.lang.String,Interval> getFluxConstraints()
```

:   Returns all current flux constraints.

    :   **Returns:**: reaction names mapped to their respective constraint.

---


### getRxnExprConstraint

```
Interval getRxnExprConstraint(MathExpr<java.lang.String> rxnNameExp)
```

:   Returns the current constraint on the provided expression.

    :   **Parameters:**: `rxnNameExp` - the mathematical expression of reaction names whose constraint is to be returned. **Returns:**: the current constraint on the provided expression.

---


### getFluxConstraint

```
Interval getFluxConstraint(java.lang.String rxnName)
```

:   Returns the current flux constraint on the reaction with the provided
    name.

    :   **Parameters:**: `rxnName` - the name of the reaction whose flux constraint is to be returned. **Returns:**: the current flux constraint on the reaction with the provided name.

---


### setFluxConstraint

```
void setFluxConstraint(java.lang.String reactionName,
                       Interval c)
```

:   Changes the flux constraint of the reaction with the provided name.

    :   **Parameters:**: `reactionName` - the name of the reaction whose flux constraint is to be changed.: `c` - the new constraint.

---


### setFluxConstraints

```
void setFluxConstraints(java.util.Map<java.lang.String,Interval> fluxConstraints)
```

:   Changes the flux constraints on more than one reaction.

    :   **Parameters:**: `fluxConstraints` - reaction names mapped to their respective flux constraints.

---


### setRxnExprConstraint

```
void setRxnExprConstraint(MathExpr<java.lang.String> e,
                          Interval c)
```

:   Changes the constraint on the provided mathematical expression
    of fluxes.

    :   **Parameters:**: `e` - a matheamtical expression of reaction names.: `c` - the new constraint.

---


### setRxnExprConstraints

```
void setRxnExprConstraints(java.util.Map<? extends MathExpr<java.lang.String>,Interval> constraints)
```

:   Changes the constraints on more than one mathematical expression
    of fluxes.

    :   **Parameters:**: `constraints` - mathematical expressions of reaction names mapped to their new respective constraints.

---


### restoreFluxConstraints

```
void restoreFluxConstraints()
```

:   Restores the flux constraints for all reactions in the fluxome to those
    values contained in the fluxome.

---


### restoreFluxConstraint

```
void restoreFluxConstraint(java.lang.String reactionName)
```

:   Restores the flux constraint for the reaction with the provided
    name to the value contained in the fluxome.

    :   **Parameters:**: `reactionName` - the name of the reaction whose flux constraint is to be restored.

---


### restoreFluxConstraints

```
void restoreFluxConstraints(java.util.Collection<java.lang.String> reactionNames)
```

:   Restores the flux constraints for the provided reaction names to the
    values contained in the fluxome.

    :   **Parameters:**: `reactionNames` - the reaction names whose flux constraints are to be restored.

---


### restoreRxnExprConstraints

```
void restoreRxnExprConstraints()
```

:   Restores the constraints on all flux expressions to the values
    contained in the fluxome.

---


### restoreRxnExprConstraint

```
void restoreRxnExprConstraint(MathExpr<java.lang.String> rxnNameExp)
```

:   Restores the constraint on the provided flux expression to the value
    contained in the fluxome.

    :   **Parameters:**: `rxnNameExp` - the expression of reaction names whose contraint is to be restored.

---


### restoreRxnExprConstraints

```
void restoreRxnExprConstraints(java.util.Collection<? extends MathExpr<java.lang.String>> rxnNameExps)
```

:   Restores the constraints on the provided expressions to the values
    contained in the fluxome.

    :   **Parameters:**: `rxnNameExps` - the reaction name expressions whose contraints are to be restored.


---


|  |  |  |
| --- | --- | --- |
| **Overview** \| **Package** \| **Class** \| **Use** \| **Tree** \| **Deprecated** \| **Index** \| **Help** | |  |
| **PREV CLASS**   **NEXT CLASS** | **FRAMES**    **NO FRAMES**     **All Classes** |  |
| SUMMARY: NESTED \| FIELD \| CONSTR \| METHOD | DETAIL: FIELD \| CONSTR \| METHOD |  |


---
